# MicroRNA Antagonism of the Picornaviral Life Cycle: Alternative Mechanisms of Interference

**DOI:** 10.1371/journal.ppat.1000820

**Published:** 2010-03-19

**Authors:** Elizabeth J. Kelly, Elizabeth M. Hadac, Bryan R. Cullen, Stephen J. Russell

**Affiliations:** 1 Department of Molecular Medicine, Mayo Clinic College of Medicine, Rochester, Minnesota, United States of America; 2 Department of Molecular Genetics and Microbiology, Duke University Medical Center, Durham, North Carolina, United States of America; University of California San Francisco, United States of America

## Abstract

In addition to modulating the function and stability of cellular mRNAs, microRNAs can profoundly affect the life cycles of viruses bearing sequence complementary targets, a finding recently exploited to ameliorate toxicities of vaccines and oncolytic viruses. To elucidate the mechanisms underlying microRNA-mediated antiviral activity, we modified the 3′ untranslated region (3′UTR) of Coxsackievirus A21 to incorporate targets with varying degrees of homology to endogenous microRNAs. We show that microRNAs can interrupt the picornavirus life-cycle at multiple levels, including catalytic degradation of the viral RNA genome, suppression of cap-independent mRNA translation, and interference with genome encapsidation. In addition, we have examined the extent to which endogenous microRNAs can suppress viral replication in vivo and how viruses can overcome this inhibition by microRNA saturation in mouse cancer models.

## Author Summary

Virus host range is shaped by cellular determinants such as transcription factors and receptor expression. In addition, we have previously shown that tissue-specific microRNAs can be utilized to direct the specificity of a replication competent picornavirus, Coxsackievirus A21. In this report, we demonstrate the mechanism by which microRNAs are able to directly influence oncolytic viruses, an important class of anticancer agents. We show that microRNA expression is an important determinant of permissivity to oncolytic virus replication, but the actual abundance of that expression is far more important. In addition, we show that there are actually multiple different stages in the life cycle of a replication competent picornavirus that are amenable to regulation by cellular microRNAs. We proceed to illustrate that microRNAs can regulate virus tropism in vivo, but demonstrate that circulating high viral titers in the blood can overcome this mechanism of conferring tissue specificity. MicroRNAs are well known to have both oncogenic or oncosuppressive activities in human cancers. Here, we show that tissue-specific microRNA expression can also be used to modulate the efficacy of viral anticancer therapeutics, and the mechanism by which they are able to do so.

## Introduction

MicroRNAs (miRNAs) are a class of small, ∼22 nt regulatory RNAs that modulate a diverse array of cellular activities. Through recognition of sequence complementary target elements found most often in the 3′UTR of cellular mRNAs, miRNAs post-transcriptionally regulate numerous cellular processes by way of mRNA translation inhibition or, less commonly, by catalytic mRNA degradation. It is thought that upwards of one-third of all human mRNAs are regulated by the over 700 human miRNAs that are currently known [1],[2]. Many miRNAs can have tissue-specific localizations and, in addition, some are now known to have cancer-specific signatures. Cancer-specific miRNAs can be both oncogenic and oncosuppressive, and growing evidence now indicates that certain miRNAs are also involved in disease progression, through the promotion of metastasis [3].

The mechanisms by which a miRNA regulates a given mRNA are influenced by parameters such as the degree of sequence homology [4] and target site multiplicity [5] as well as by features of the mRNA itself, including target site secondary structure [6] and location [7]. In addition, the cellular machinery used to translate mRNAs is thought to profoundly affect miRNA regulation. While capped mRNAs are known to be amenable to both catalytic miRNA-induced cleavage and miRNA-mediated translational repression, it has been suggested that uncapped mRNAs that rely on an IRES (Internal Ribosome Entry Site) for translation initiation are not susceptible to translational repression [8] [9].

In addition to their roles in the pathogenesis of human disease, endogenous cellular miRNAs can also play a role in viral infection, acting to suppress [10] [11] [12] [13] or enhance [14] viral replication. Recently, miRNAs have been exploited to influence the tissue tropism and pathogenicity of viruses used as vaccines [10], anticancer therapeutics [13], and gene transfer vehicles [15]. MiRNAs of both cellular and viral origin are thought to be involved in regulating the host response to viral infection [16] and miRNAs have been shown to regulate viral antigen presentation [17], the antiviral interferon response [18], viral tissue tropism and antiviral immunity [19].

Engineering miRNA-responsiveness in viruses used as cancer therapeutics has been shown to be an effective way to generate tumor selectivity [19]. And although miRNAs clearly play a role in multiple aspects of both the viral replication cycle and the host response to viral pathogens, little is known about the mechanisms by which these regulatory molecules act to directly influence the level of virus replication. To this end, we engineered the oncolytic picornavirus Coxsackievirus A21 (CVA21), to encode artificial miRNA targets complementary to cellular miRNAs. Here, we look at the ability of these miRNA targets to restrict viral replication in cells expressing cognate miRNAs and the mechanisms by which they are able to do so. By utilizing CVA21 derivatives bearing different miRNA target elements (miRTs), including targets with varying degrees of homology to endogenous cellular miRNAs and targets in orientations designed to target either the positive-strand RNA genome or the negative-strand antigenome, we were able to identify multiple, distinct steps in the picornaviral life cycle that are amenable to miRNA-mediated regulation.

CVA21 is a picornavirus known to cause upper respiratory and inflammatory muscle infections in humans [20] [21]. It replicates quickly and efficiently in both cell culture and in mouse models, and is a typical positive-strand RNA virus. The genome of CVA21 is a single-stranded, uncapped, positive-strand RNA, which is translated to yield a polyprotein that is then cleaved by viral proteases to form the viral capsid and nonstructural proteins. We have previously reported that CVA21 has potent oncolytic activity against a variety of human cancers, and can mediate complete tumor regression in mice bearing human melanoma or myeloma xenografts [13]. However, this potent and curative oncolytic activity is accompanied by a high-level viremia and rapid-onset lethal myositis that hampers the feasibility of employing this virus as an anticancer therapeutic in the clinic.

Incorporation of miRNA target elements (miRTs) corresponding to two muscle-specific cellular miRNAs (miR-133 and miR-206) was shown to mediate silencing of CVA21 gene expression in cells expressing muscle-specific miRNA mimics, in muscle culture lines and, most importantly, in vivo in mice. Cells expressing exogenous or endogenous miRNAs complementary to miRTs are intrinsically immune to infection by the engineered CVA21 and are protected from the cytolytic effects of this virus. Thus, tumor-bearing mice infected with a recombinant muscle-restricted virus (CVA21 miRT) were cured of established tumors whilst being protected from productive muscle infection and the resultant myositis. Our ability to engineer the oncolytic picornavirus CVA21 to contain diverse miRTs provided an opportunity to look at the mechanisms by which cellular miRNAs can act directly on a virus to silence gene expression and mediate tumor selectivity.

## Results

### MicroRNAs can recognize and regulate Coxsackievirus A21

**Figure 1:**
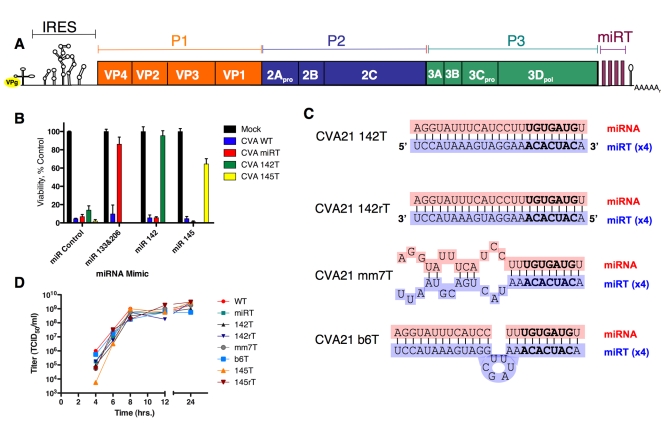
Schematics, growth kinetics, and permissivity of recombinant CVA21 in HeLa cells. (A) Schematic diagram of microRNA insertion in CVA21. (B) Viability of HeLa cells transfected with indicated miRNA-mimics 48 hours post infection with indicated recombinant CVA21. (C) Schematic overview of miRNA/target homology of recombinant inserts. (D) One -step growth curves of recombinant CVA21s on HeLa cells.

To investigate at what stage(s) of the viral life cycle cellular miRNAs are able to perturb viral replication, we utilized our previous recombinant miRT design whereby four tandem copies of a given target element corresponding to a cellular miRNA are incorporated into the 3′UTR of the viral genome (Figure 1A). In order to identify a cellular miRNA that could effectively inhibit CVA21, we engineered the CVA21 genome to encode tandem miRTs complementary to four different tissue-specific miRNAs. Target elements corresponding to muscle-specific (miR-133 and miR-206), hematopoetic-specific (miR-142-3p) or tumor-suppressor (miR-145) miRNAs were incorporated in the 3′UTR of CVA21 and protection of HeLa cells transfected with sequence-complementary miRNA mimics (synthetic dsRNAs corresponding to cellular miRNA duplex intermediates) was analyzed (Figure 1B). While miRNA-mimics were able to protect cells infected with viruses containing sequence complementarity from ∼60% to levels that were not significantly different from control, the hematopoetic-specific miR-142-3p target provided the most complete and most consistent protection, and hence provided the best candidate to study the different steps of virus replication at which cellular miRNAs could act to perturb viral replication.

To identify miRNA-mediated antiviral activity acting at distinct steps in the viral life cycle, we designed a panel of recombinant viruses expressing variations of the miR-142-3p target. Four tandem fully complementary sites were designed to provide the best opportunity for direct catalytic cleavage of the viral RNA genome and mRNA (CVA21 142T); four copies in reverse orientation were designed to address the existence of miRNA-mediated antiviral activity acting on the negative-sense antigenome (CVA21 142rT). In addition, two viruses were constructed to look at the potential for translational silencing of the viral mRNA: a virus containing 7 base pairs of mismatch between the target and miR-142-3p (CVA21 mm7T) and a virus that contained the entire miR-142-3p target site with a 6 base pair (bp) stuffer sequence inserted between bp 10/11 of the target site (CVA21 b6T), each with four replicate copies (Figure 1C). The latter, or ‘bulge’, virus contained extra sequence at the catalytic cleavage site to provide a strong candidate for miRNA-mediated translational suppression: four perfect copies of the miRT to promote miRNA recognition, and a “stuffer sequence,” at the site at which the RNA induced silencing complex (RISC) ‘slicer’ activity cleaves (to block catalytic degradation of the viral RNA). These recombinant viruses were rescued in the absence of the corresponding miRNAs and found to replicate to high titer with growth kinetics analogous to the wild-type (WT) virus in cells lacking miR-142-3p (Figure 1D).

### Translational silencing of CVA21 mRNA

Cellular microRNAs are known to mediate post-transcriptional silencing of sequence-complementary mRNAs through translational repression and/or transcript degradation. However, in the absence of a 5′ cap, and in the presence of the IRESes utilized by many viruses, it has been hypothesized that miRNAs are unable to induce translational repression [22]. These studies have been performed primarily with reporter mRNAs containing one of several viral IRESes and generally in the presence of short interfering RNAs (siRNAs), cell free extracts, or miRNA mimics supplemented in trans [8],[23] [24].

Translation of the CVA21 mRNA into the viral polyprotein is mediated by a type I IRES in a cap-independent manner. We reasoned that the analysis of recombinant CVA21 variants targeted by miRNAs might be particularly useful in studying the translational silencing of cap-independent mRNAs because cells can be infected at very low multiplicities of infection (MOI), thus circumventing the potentially confounding issue of miRNA saturation [25], and enabling the quantitative analysis of virus expansion whilst simultaneously allowing precise measurement of cell death at specific times post-infection.

**Figure 2:**
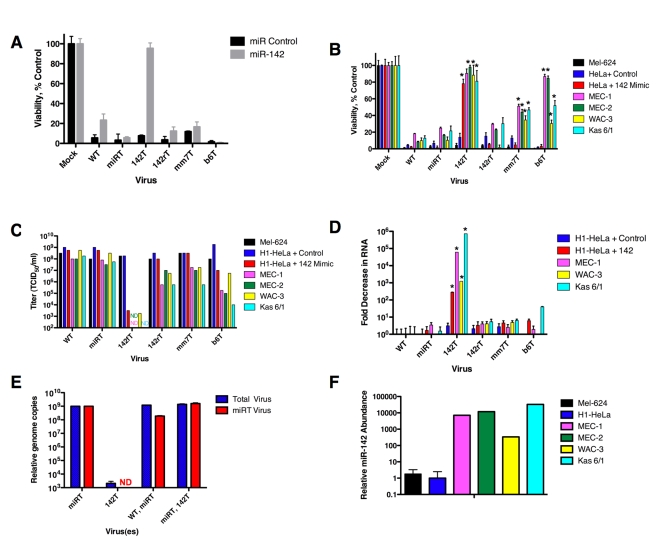
Translational suppression of CVA21 occurs in the presence of high endogenous miRNAs. (A) Viability 48 hrs. post infection with MOI = 1.0 recombinant CVA21s in the presence of 200 nM control or miR-142 mimic. (B) Viability 48 hrs. post infection with MOI = 1.0 in a panel of hematopoetic or control cell lines or in H1-HeLa cells transfected with 200 nM control or miR-142-3p mimics. (C) Infectious titer in cell supernatant in (B), titrated on H1-HeLa cells and assayed 72 hrs. post infection. (D) Fold decrease in CVA21 RNA 48 hrs. post infection at MOI = 1.0 of hematopoetic cell lines or in trans supplemented miRNA mimic. Fold decrease normalized to GAPDH expression. (E) Relative CVA21 genome copies in singly or doubly infected MEC-1 cells as quantified by qPCR. Primers and probes in 2A region of CVA21 or miRT region of CVA21 genome quantified total or miRT viral genomes, respectively. (F) Relative miR-142-3p abundance in a panel of cell lines. Small RNA was extracted from cells and reverse-transcription and qPCR analysis of miR-142 3p was performed. Abundance correlated to expression in H1-HeLa cells (per 5 ng small RNA). *p = <.01 vs. H1-HeLa + Control; ND- none detected.

Similar to experiments conducted by other groups [9] [23], we found that in the presence of miRNA mimics added in trans, viruses containing multiple imperfectly complementary target sites were unresponsive to miRNA-mediated repression and cells were not significantly protected from viral cytopathic effects (Figure 2A). Increasing miRNA mimic copy numbers did not cause any increase in protection from cytolysis by these viruses (data not shown). However, hematopoetic cell lines and primary hematopoetic cells expressing high levels of endogenous miR-142-3p were significantly less susceptible to viruses containing mismatch (mm7T) or bulge (b6T) targets (Figure 2B; p<0.01 and p<0.004, respectively). In both cases, viral titers were reduced by up to 5 orders of magnitude (Figure 2C). Although targeting the viral antigenome had no significant effect on cell viability, this did reduce viral titers by up to 100 fold (Figures 2B and 2C).

To determine whether the observed increase in cell viability was indeed as a result of translational silencing rather than transcript cleavage, we looked at CVA21 RNA copy numbers. A panel of hematopoetic cells, as well as H1-HeLa cells (serving as a control cell line) were supplemented with control or miR-142 mimics, and all cells were infected with the panel of recombinant CVA21 viruses. Total cellular RNA was harvested and viral RNA was quantified and normalized to cellular GAPDH mRNA levels. While inserting perfectly complementary miR-142-3p targets (in CVA21 142T) reduced CVA21 RNA levels by up to a million-fold, the mismatched CVA21 mm7T and bulged CVA21 b6T targets induced only a modest decrease in viral RNA (Figure 2D). Translational silencing would be expected to indirectly cause a small drop in viral RNA levels through production of less viral polymerase, presumably accounting for the up to 40 fold decrease in CVA21 RNA in the presence of bulged targets (Figure 2D).

To test whether CVA21 142T RNA was reduced in the presence of cells bearing sequence-complementary miRNAs due to direct cleavage of the viral RNA, or rather because translational silencing had reduced the amount of viral polymerase to amplify the viral RNA, we performed a trans-complementation experiment. Hematopoetic MEC-1 cells were infected with two different CVA21s: miRT CVA21 (containing muscle-targets), and CVA21 142T (containing hematopoetic targets). In MEC-1 hematopoetic cells, CVA21 142T should be miRNA-targeted, while miRT CVA21 should replicate unencumbered. qRT PCR was then performed to ascertain the total amount of viral RNA in the cells, and what portion of it was miRT CVA21 (the difference between total CVA21 RNA and miRT CVA21 RNA would therefore be RNA corresponding to CVA 142T in cells doubly infected with miRT CVA21 and CVA21 142T). Complementation by miRT CVA21 polymerase should be capable of synthesizing CVA21 142T if any remained that had not been catalytically destroyed, (however the efficiency of trans-complementation in picornaviruses still remains under debate [26] [27] [28]).

Quantitative PCR analysis revealed that all virus present in singly infected miRT CVA21 analysis was indeed from miRT CVA21 (Figure 2E). MEC-1 cells infected with only CVA21 142T had a larger than 5 log decrease in total CVA21 RNA (and, as expected did not contain any miRT CVA21). In cells doubly infected with WT+ miRT virus, less than 50% of progeny genomes were found to be miRT genomes (the remainder are therefore WT progeny genomes). However, in cells infected with CVA21 142T + miRT CVA21, 100% of progeny genomes were found to be miRT genomes (none were therefore 142T genomes). Hence, 142T genomes are destroyed and not amplified in cells containing the 142T miRNA, even when the cells are infected with another virus that provides all viral proteins in trans.

While we observed that the CVA21 142T RNA genome was profoundly decreased in the presence of perfectly matched miR-142 mimics, there was significant variability in the degree of inhibition seen in different hematopoetic cell lines (Figure 2D). To determine whether this variability correlated with the level of miR-142-3p expression in these cells, we analyzed the abundance of endogenous miR-142-3p in our panel of hematopoetic cell lines by qRT-PCR. The chronic lymphocytic leukemia (CLL) line MEC-1 and the multiple myeloma cell line Kas 6/1 expressed the highest levels of endogenous miR-142-3p (Figure 2F), and showed the greatest inhibition of CVA21 142T RNA expression (Figure 2D), followed by the CLL lines MEC-2, MEC-1, and WAC-3. We observed an inverse correlation between miR-142-3p expression levels and the CVA21 142T RNA copy numbers detected, suggesting that the abundance of this particular miRNA contributes substantially to the efficiency with which it acts to silence complementary mRNAs.

Overall these results show that perfectly matched miR-142 targets are recognized and catalytically degraded. In addition, while clearly not as efficient as mRNA cleavage, translational repression was seen to occur with cap-independent type I IRES-containing mRNAs and provided protection from cytopathic effects mediated by the virus, and the reduced the infectious virus released into the cellular supernatant. This translational silencing was only detected in the presence of high levels of endogenous miRNAs, however, and at a low multiplicity of viral infection.

### Cellular microRNAs perturb the picornaviral life cycle at multiple steps

Having determined that viral replication was amenable to miRNA-mediated regulation at two of the earliest steps in viral infection (genome cleavage after uncoating and initial mRNA translation), we next sought to look at later steps in the viral life cycle. While perturbing the virus life cycle at early steps after infection (attacking genome after uncoating, initial mRNA expression) would be most likely to slow or inhibit a productive infection, later steps such as mRNA accumulation, the bulk of mRNA and genome accumulation, and encapsidation of the viral genome could be equally amenable to miRNA-mediated regulation as exposed targets are still present on viral RNAs. In order to examine the ability of microRNAs to act on late steps of viral replication, we conducted a time-course experiment where miRNA mimics were added prior to, concomitant with, or at specific times after infection.

Picornaviruses are known to inhibit host cell translation beginning at about 1 hour after infection [29], with viral mRNA accumulation peaking by 5 hours post infection [30], followed by encapsidation of the positive-strand viral RNA into the provirion and cytolytic release thereafter [31]. In order to present blocks at different stages of the picornaviral life cycle, we therefore infected cells either pre-treated with miRNA mimic, or mimics were added at 0, 3, or 6 hours post infection (pi).

**Figure 3:**
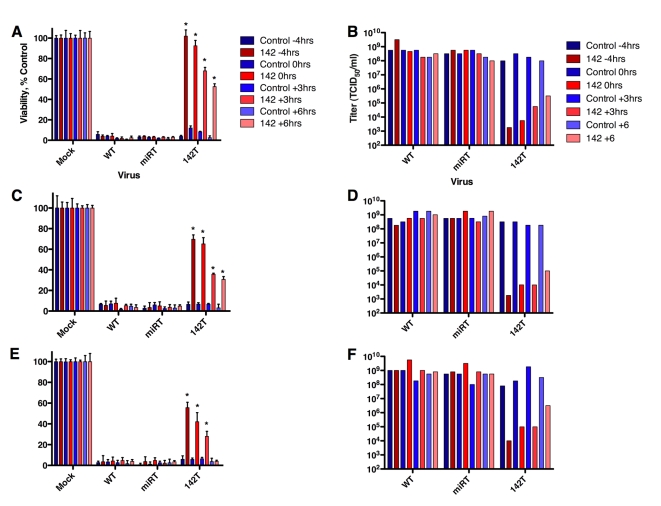
MicroRNAs inhibit late-stage picornavirus infections. Viability 48 hrs. post infection with (A) MOI = 1.0, (C) MOI = 3, (E) MOI = 10 CVA 142T in the presence of control or miR-142-3p mimic added 4 hours prior to infection, or at 0, 3, or 6 hours post infection. (B) Viral yield from cell supernatant as measured by TCID_50_ assay from (A) on H1-HeLa cells. (D) Viral yield from cell supernatant as measured by TCID_50_ assay from in (C) on H1-HeLa cells. (F) Viral yield from cell supernatant as measured by TCID_50_ assay from (E) on H1-HeLa cells. *p = <.01 (as compared to H1-HeLa + Control).

**Figure 4:**
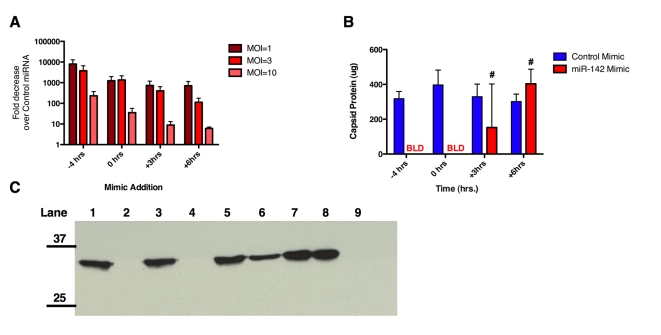
MicroRNAs can affect particle to infectivity ratio of CVA21. (A) Fold change in CVA21 142T RNA as determined by qRT-PCR of viral RNA from supernatant of infected cells at indicated time pre/post miRNA mimic addition. Fold decrease expressed as ratio of CVA 142T RNA in the presence of miR-142 mimic/CVA 142T RNA in the presence of control miRNA mimic (at indicated MOI). (B) Protein assay of sucrose-purified virus collected in the presence of miRNA mimics added at 4 hours prior, or 0, 3, or 6 hours after virus infection at MOI = 1. (C) Western blot with anti-CVA21 of sucrose-purified supernatant run on 12.5% SDS-PAGE gel from miRNA mimic timecourse at MOI = 1. Lane 1-Control mimic -4 hrs pi + CVA 142T; Lane 2- 142 mimic -4 hrs pi + CVA 142T; Lane 3- Control mimic 0 hrs pi + CVA 142T; Lane 4- 142 mimic 0 hrs pi +CVA 142T; Lane 5- Control mimic +3 hrs pi + CVA 142T; Lane 6- 142 mimic +3 hrs pi + CVA 142T; Lane 7- Control mimic +6 hrs pi + CVA 142T; Lane 8- 142 mimic +6 hrs pi +CVA 142T; Lane 9- Mock infected. BLD- below limit of detection. #p = ns (vs. H1-HeLa + Control mimic). pi: post infection.

Analysis of cell viability in the presence of miRNA mimics added at successive times after infection demonstrated that, while mimic presence prior to infection provides the greatest protection from viral cytolysis, miRNAs can negatively impact viral replication such that cell viability is protected by nearly 50% even when added 6 hours after infection (Figure 3A). Moreover, production of viral progeny is greatly diminished in the presence of miRNAs added at varying time points up to 6 hours post-infection (Figure 3B). To confirm that this was not an artifact of virus expansion (ie cells merely being protected by virtue of not being infected until one round of viral replication had occurred), this experiment was repeated at an MOI = 3 or 10 and similar results observed (Figure 3C–3F). In addition, we analyzed the quantity of CVA21 142T by qRT-PCR in the presence of control miRNA mimics as compared to CVA21 142T infection in the presence of miR-142-3p mimic at different MOI (Figure 4A) and found that virus abundance was similar to that observed when analyzed through infectious titer (Figure 3B, 3D, 3F).

Since the picornavirus life cycle is particularly rapid and the bulk of viral RNA amplification, protein expression and provirion production has already taken place by 6 hours after infection, we wondered whether miRNA mimics might be interfering with genome encapsidation. We therefore sought to look at the particle: infectivity ratio of the released progeny viruses in the presence of miRNA mimics added after viral infection. Infectivity was determined by titration of cell supernatant on H1-HeLa cells while capsid protein content was assayed after concentration through sucrose cushions of the supernatant of infected cells by both protein assay and Western analysis. Capsid proteins were not detected in sucrose-purified samples of cellular supernatant to which sequence-complementary mimics (miR-142-3p) were added before or simultaneously with CVA21-142T infection (Figure 4B). However, when miR-142-3p mimic was added 3 or 6 hours after virus infection, total capsid protein was not significantly diminished as compared to cells transfected with a control miR (p = 0.3, p = 0.14, Figure 4B). Infectious units, however, were decreased by 4 and 3 logs, respectively (Figure 3B). Similarly, when these sucrose-purified fractions were subjected to Western analysis with antibodies raised against CVA21, CVA21 protein was roughly equivalent when CVA 142T infection was performed with in the presence of control miRNA mimics (Figure 4C). However, miR-142-3p mimic present 4 hours prior to infection or simultaneously with CVA 142T infection inhibited detectable expression of CVA21 capsid in the immunoblot. In contrast, when miR-142-3p mimic was added at either 3 or 6 hours after expression, accumulation of viral capsid was detected in Western blots of the sucrose-purified portion and corresponded with capsid quantified by protein assay.

**Figure 5:**
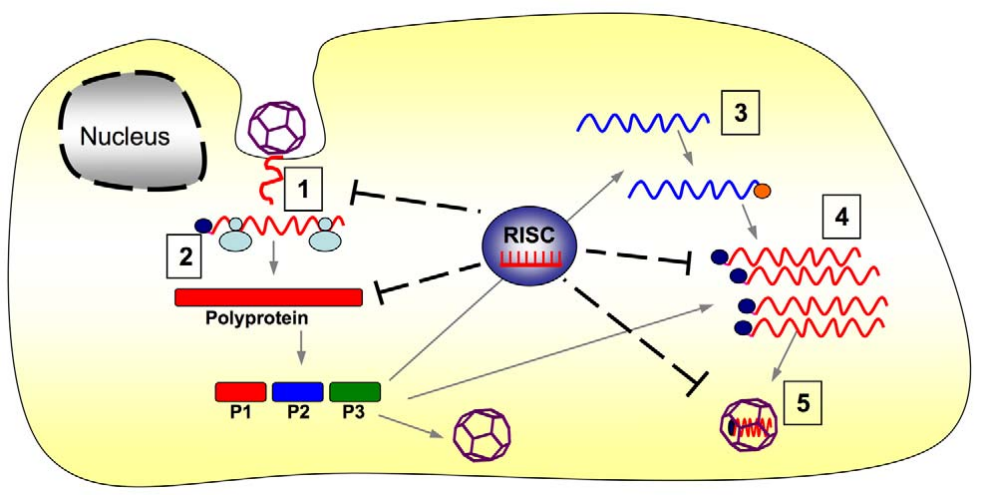
RISC-mediated inhibition of CVA21 life cycle. Stages in the picornaviral life cycle at which miRNAs could potentially act: 1-genome attack after uncoating; 2-polyprotein translation; 3-negative strand RNA synthesis; 4- positive strand RNA accumulation; 5-encapsidation.

Together these results reveal that alternative steps in the picornaviral life cycle are amenable to miRNA-mediated inhibition: including (but perhaps not limited to) RNA cleavage after uncoating, reduced translation of the viral mRNAs, reduced viral mRNA accumulation, and diminished encapsidation of the viral genome into the procapsid (Figure 5).

### MicroRNAs *in vivo*: saturation and selective pressure

While the in vitro determination of the mechanisms by which miRNAs are able to inhibit virus replication can facilitate the design of better and more elegant ways to exploit cellular miRNAs for gene transfer and vaccine development for multiple virus families, in vivo evaluation is necessary to validate this targeting paradigm for translational purposes, and to demonstrate that it will indeed occur in model organisms.

**Figure 6:**
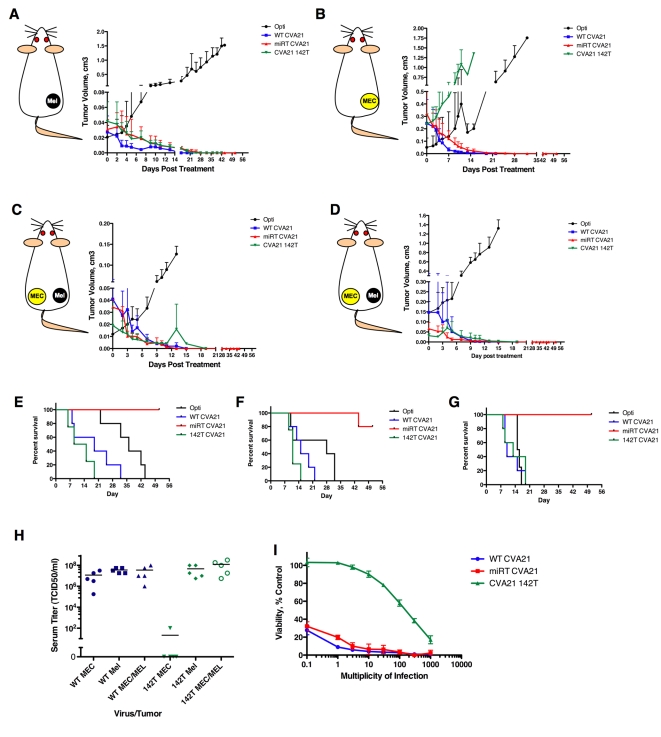
MicroRNAs regulate virus tropism in vivo, but can be saturated by high-level viremia. (A) SCID mice bearing Mel-624 xenografts were treated with one intratumoral dose of 1×10^6^ recombinant CVA21 and tumors measured daily. (B) SCID mice bearing MEC-1 xenografts were treated with one intratumoral dose of 1×10^6^ recombinant CVA21 and tumors measured daily. (C) SCID mice bearing bilateral Mel-624 and MEC-1 xenografts were treated with one intravenous dose of 1×10^6^ recombinant CVA21 and Mel-624 tumors measured daily. (D) SCID mice bearing bilateral Mel-624 and MEC-1 xenografts were treated with one intravenous dose of 1×10^6^ recombinant CVA21 and MEC-1 tumors measured daily. (E) Kaplan-Meier survival graphs of mice in (A). (F) Kaplan-Meier survival graphs of mice in (B). (G) Kaplan-Meier survival graphs of mice in (C,D). (H) Virus titer in mouse serum in mice from (A–D). (I) In vitro saturation of miR-142 as determined by MTT assay in MEC-1 cells infected with rCVA21 at MOI .1-1000.

Therefore, we next sought to confirm that tumor cells expressing endogenous miRNAs were capable of targeting the CVA21 genome and would indeed protect from viral replication in vivo in the mouse model. To this end, 5×10^6^ Mel-624 (melanoma) or MEC-1 (chronic lymphocytic leukemia, CLL) cells were implanted subcutaneously in immunodeficient mice. When tumors reached an average of 250 mm^3^ in size, the animals were treated with a single intratumoral dose of 10^6^ TCID_50_ of a recombinant CVA21, after which time tumor growth and survival were monitored (Figure 6).

As expected, all CVA21 variants (WT, miRT and142T) replicated in the melanoma-xenografts (which express no sequence-complementary miRNAs) such that complete (or near complete) tumor regression took place (Figure 6A). Animals receiving the WT or 142T viruses rapidly succumbed to myositis, necessitating euthanasia (Figure 6E). Also as expected, animals treated with our previously described muscle-restricted virus (miRT) had complete tumor regressions and were protected from the development of myositis, while Opti-MEM injected control tumors grew unencumbered and mice had to be sacrificed when tumors reached ∼2.0 cm^3^. In contrast to the melanoma xenografts, MEC-1 CLL xenografts (which express abundant miR-142) were susceptible to the WT and muscle-restricted viruses but were highly resistant to oncolysis by CVA21 142T (Figure 6B).

Since all recombinant viruses behaved as expected in mice bearing one susceptible or one restricted tumor type, we next decided to examine their behavior in animals with a different tumor on each flank after intravenous virus administration. By using contralateral tumors we were able to create a more representative in vivo scenario, with multiple CVA21-permissive tissues having distinct (permissive or restrictive) miRNA expression profiles. Unexpectedly, the virus bearing tandem repeats of miR-142 (CVA21 142T) replicated in both the permissive melanoma xenograft (Figure 6C) and in the “nonpermissive” CLL xenograft (Figure 6D) in these mice, such that both tumors regressed just as rapidly as when they were infected with the nontargeted WT or miRT viruses. Once again, only the muscle restricted virus (miRT CVA21) prolonged the survivals of tumor-bearing mice, (Figure 6E p = 0.0018, Figure 6F p = 0.0004, Figure 6G p = 0.0062).

To confirm that CVA 142T was indeed replicating in both the melanoma and myeloma xenografts, both tumors were explanted from two separate mice at time of euthanasia. Tissue was homogenized and overlaid on H1-HeLa cells to look for viral recovery. In each tumor of both animals, virus was recovered and found to contain no alteration in the 142T sequence, indicating that there was indeed productive infection in both tumors.

In order to determine why regression of mir-142-expressing hematopoetic tumors was observed in animals bearing contralateral melanoma xenografts when treated with the miR-142 restricted virus, but not in animals with a single hematopoetic tumor, we compared the viral titers in serum from animals treated with CVA21 142T versus WT CVA21 (Figure 6H). Serum titers were typically high in mice treated with WT CVA21, irrespective of whether they were implanted with melanoma, CLL or bilateral xenografts. In mice treated with CVA-142T, the serum titers were very high (>10^8^) in animals bearing melanoma xenografts, very low (<10^2^) in those with hematopoetic tumor xenografts, and were again very high in animals with the combination of melanoma and hematopoetic xenografts on either flank.

Since serum titers were very high in mice with bilateral tumors (>10^8^ TCID_50_/ml), we investigated the possibility that endogenous miR-142 expressed in MEC-1 xenografts could have been saturated by this high-level viremia. To investigate this possibility, we conducted an in vitro assay looking at saturation of endogenous miR-142 in MEC-1 cells. While cells were completely protected against cytotoxicity at MOIs of up to 30, increasing virus titer thereafter resulted in decreased cell viability such that by an MOI = 1,000 we saw near complete cytopathic effects in CVA21 142T infected cells (Figure 6I), thus suggesting that the endogenous miR-142 may have been effectively saturated, a phenomenon which has been observed by a number of groups previously [32] [25]. While our in vitro data suggest that a sequence-specific saturation of endogenous miRNAs is possible, other possibilities include a non-specific shutdown of all RNAi machinery in infected cells, or possibly the destruction of tumor vasculature that serves as a necessary scaffolding for xenografts.

Together these data show that miRNAs act *in vivo* to restrict virus tropism in cells permissive to viral replication. Perhaps not surprisingly, we found that persistent high-level viremia was able to overcome this miRNA-mediated restriction, possibly through allowing the saturation of endogenous miRNAs for which our virus bore tandem cognate targets. We speculate that in animals bearing bilateral tumors treated with intravenous CVA21 142T, virus trafficked to the susceptible melanoma tumor, which created a reservoir of viral amplification. This then resulted in a high-level viremia, which was able to overwhelm the miRNA-restricted CLL tumor by saturating the sequence-complementary endogenous miRNA. We detected no sequence alteration in the virally encoded miRT insert in animals treated with miRT or CVA21 142T, and therefore can dismiss the possibility that the viruses overcame miRNA restriction in this manner. However, we did identify significant sequence insert changes in viruses bearing the tumor suppressor miR-145T inserts when administered to this same panel of animals (data not shown). We therefore surmise that miRNA targets are differentially effective and susceptible to different selective pressures to mutationally inactivate these targets, and that this may correspond to the specific tissues from which they are being excluded.

## Discussion

Increasing insights into the interactions between miRNAs and viruses have suggested new therapeutic targets for antiviral drugs and new techniques for the creation of vaccines, as well as for regulating the tissue specificity of gene transfer vehicles and oncolytic viruses. However, the ways in which cellular miRNAs can act to inhibit viral replication have remained ill-defined. In this report, we identify alternative steps in the viral life cycle that are amenable to miRNA-mediated inhibition in the context of a replication-competent oncolytic virus that acts as a fully curative therapy in xenograft-bearing mice.

Here, we show that picornaviruses are most susceptible to miRNA-mediated attack immediately after infection, and that the viral RNA genome can be recognized and cleaved by fully sequence complementary miRNAs. However, contrary to the currently accepted paradigm, we also present evidence to suggest that miRNAs can translationally suppress uncapped, IRES-dependent mRNAs bearing imperfectly matched target sequences at low multiplicity of infection. Several previous studies have used the tumor-suppressor miRNA let-7a to look at miRNA regulation in cap dependent vs. IRES dependent mRNAs. However, despite very high let-7a abundance in a number of cell lines, target elements corresponding to let-7 do not functionally suppress sequence complementary targets as efficiently as does miR-142 (or a number of other well-characterized miRNAs) [5]. In addition, the interpretation of data generated using cell-free extracts and synthetic miRNAs supplemented in trans, as employed previously by several groups, may be made more difficult by miRNA saturation. We believe the use of a highly efficient miRNA target (miR-142T), cell lines expressing high levels of endogenous, sequence-complementary miRNAs, and the ability to infect cells at low MOI, have enabled us to see the subtle translational suppression of a viral mRNA in a cap-independent context.

The observation that the picornaviral life cycle can be efficiently interrupted by miRNAs even at very late stages after infection, by which time the viral genome copy number has amplified to a very high level, is truly remarkable. This late interruption appears to require perfect complementarity between the viral genome and the miRNA, and probably in large part reflects the rapid catalytic destruction of viral genomes. An interesting consequence of adding the miRNA late after infection to interrupt the viral life cycle is that the cell still releases abundant virus particles, but that they lack infectivity (Figures 3, 4). The possibility exists that miRNAs could also block encapsidation of viral RNA genomes by steric hindrance (RISC bound-RNAs being too large to be packaged), although our data do not address this possibility.

While our data show that miRNAs can recognize and antagonize a virus, our observation that endogenous cellular miRNAs can actually overwhelm this regulation is also of potential importance. The phenomenon of miRNA saturation, whereby miRNA targets can act as ‘sponges’ to bind and sequester endogenous cellular miRNAs, has been reported previously, but has been generally thought to occur in the context of translational silencing [25] [32]. Here, we show that miRNA saturation can also occur when miRNA regulation is occurring primarily by catalytic degradation of a perfectly complementary RNA target (Figure 6I), and that it can happen in the context of viral infection in vivo.

We have shown that miRNAs can suppress virus propagation in vivo and can provide post-entry blocks to virus replication in otherwise permissive cells. However, we also show that miRNA defenses can be overcome at very high multiplicities of viral infection in vivo, allowing unencumbered virus replication. Therefore, clinicians considering therapeutic intervention utilizing miRNA targeted-viruses (ie vaccines and cancer therapeutics) must be particularly wary of the possibility that unencumbered virus replication in permissive cells, eg a tumor, may eventually lead to viral infection and pathogenesis in normal cells that express a restricting miRNA (if that miRNA becomes saturated).

MiRNAs are known for their ability to regulate numerous cellular functions, often at multiple steps within a single signal cascade. In this report, we have shown that miRNAs can also act to antagonize sequence complementary viruses at multiple, alternative steps in the virus life cycle such that tumor selectivity is generated, but also a mechanism by which this can be overcome. Although we show that picornaviruses are particularly susceptible to miRNA interference, the different replication steps affected by miRNAs are common to many viruses and it is therefore likely that multiple virus families will be amenable to miRNA-mediated regulation.

## Materials and Methods

### Ethics statements

Mayo Clinic Institutional Care and Use Committee reviewed and approved protocol A7007 with investigators SJR, EJK, and EMH, entitled “MicroRNA-mediated targeting of an oncolytic enterovirus, Coxsackievirus A21”. All mice were housed in the BSL-2 facility at Mayo Clinic and experiments were performed in compliance with outlined and approved institutional guidelines.

### Cell culture

H1-HeLa, cells were obtained from American Type Culture Collection and were maintained in DMEM supplemented with 10% FBS in 5% CO_2_. MEC-1, MEC-2, WAC-3, and Kas 6/1 cells were obtained from Diane Jelenik, Dept. of Immunology, Mayo Clinic. MEC-1 and MEC-2 cells were maintained in IMDM 10% FBS in 5% CO_2_. WAC-3 and Kas 6/1 cells were cultured in RPMI with 10% FBS in 5% CO2.

### Recombinant CVA21 construction

pGEM-CVA21 clone was kindly provided by Matthias Gromeier. miRNA sequences were obtained from the Sanger Institute miRBase (http://microrna.sanger.ac.uk/sequences/). The following sequences were cloned into the 3′UTR of pGEM-CVA21 in between bp 7344/7345 by overlap extension PCR.

*miR-142 3pT*

TCCATAAAGTAGGAAACACTACA CGATTCCATAAAGTAGGAAACACTACAACCGGTTCCATAAAGTAGGAAACACTACATCACTCCATAAAGTAGGAAACACTACA

*miR-142revT*

TGTAGTGTTTCCTACTTTATGGA ATCGTGTAGTGTTTCCTACTTTATGGAACCGGTTGTAGTGTTTCCTACTTTATGGAATCGTGTAGTGTTTCCTACTTTATGGA

*miR-142 mm7T*

TTAATGCAGTCATAAACACTACA CGATTTAATGCAGTCATAAACACTACAACCGGTTTAATGCAGTCATAAACACTACATCACTTAATGCAGTCATAAACACTACA

*miR-142 b6T*

TCCATAAAGTAGGTCGATTAAACACTACA CGATTCCATAAAGTAGGTCGATTAAACACTACAACCGGTTCCATAAAGTAGGTCGATTAAACACTACATCACTCCATAAAGTAGGTCGATTAAACACTACA

*miR-145T*

AGGGATTCCTGGGAAAACTGGAC CGATAGGGATTCCTGGGAAAACTGGACACCGGTAGGGATTCCTGGGAAAACTGGACTCACAGGGATTCCTGGGAAAACTGGAC

### Virus production, titration

Viral RNA was produced using Ambion Megascript and Megaclear T7 polymerase kit according to manufacturers instructions. For rCVA21 rescue 1 ug RNA/well was transfected into H1-HeLa cells in 12 well plates using the Mirus RNA transfection reagent and at 24 hours post infection wells were scraped and cell pellets harvested. Cell pellets were subjected to 3 freeze/thaw cycles in liquid N2, cell debris was cleared by centrifugation and cleared lysate was added to H1-HeLa cells in a T-75 flask. Titration of CVA21 was performed on H1-HeLa cells. Cells were plated in 96 well plates at 50 percent confluence. After 24 hours, serial ten-fold dilutions (−2 to −10) were made of the virus; 100 uL of each dilution was added to each of eight duplicate wells. Following incubation at 37°C for 72 hours, wells were then assessed for CPE and TCID_50_ values were determined using the Spearman and Kärber equation.

### One-step growth curves

H1-HeLa cells were incubated with rCVA21 at a multiplicity of infection (MOI, determined by TCID_50_ per cell) of 3.0 for 2 hours at 37°C. Following this incubation, cells were washed and resuspended in fresh growth media at predetermined time-points (2, 4, 6, 18, 12, 24, hours), cells pellets were harvested and frozen at −80°C. At the completion of all time-points, they were thawed, and cell pellets were cleared from the samples by centrifugation providing a cleared cell lysate fraction.

### MiRNA mimics

miRNA mimics were purchased from Dharmacon, Inc. Control miRNA mimic corresponded to a C. elegans miRNA with no predicted miRTs in mammalian cells according to manufacturer. miRNA mimics were transfected with Mirus ™ RNA transfection reagent at a 200 nM concentration. 4 hours post mimic transfection, cells were infected with recombinant CVA21 at MOI = 1.0, 3, or 10, unless other time noted. After 24 hrs. post infection, cells were harvested for MTT viability assay and supernatant was harvested for titration.

### Quantitative RT-PCR

Total cellular RNA was harvested using the Qiagen RNeasy Kit, according to manufacturer instructions. Primers and probes corresponding to the 2A region or miRT insert region of CVA21 were used to quantitate CVA21 RNA and GAPDH primers and probes were used to normalize total cellular RNA. For each sample analyzed, 50 ng of total RNA was subjected to qRT-PCR in triplicate on Stratagene Mx4000 qPCR system using the Taqman One Step RT PCR master mix.

### MicroRNA analysis

Small RNAs were harvested from all cell lines with the Ambion miRVana microRNA isolation kit, according to manufacturer instructions. RNA was resuspended in 50 ul nuclease free water, and quantified by spectrophotometer. For analysis of miRNA expression, the Applied Biosystems Taqman microRNA Assay system was used. For each miRNA analyzed, 5 ng of small RNA was subjected to qPCR in triplicate on Stratagene Mx4000 qPCR system.

### Viral particle quantification

Ten-centimeter dishes of H1-HeLa cells were transfected with miRNA mimics and infected with CVA21 142T at MOI = 1.0 at indicated time points. Cell supernatant was collected centrifuged for 5 mins at 10,000 g to clear cell debris. Infectious titer was calculated on cell supernatant, and thereafter .5% SDS and 2 mM EDTA was added to supernatant, and then overlaid on a 5 ml sucrose cushion. Total virus particles were subjected to ultracentrifugation for 4 h at 28,000 rpm using an SW28 swinging bucket rotor. Supernatants were discarded and centrifuge tubes were rinsed with PBS and re-spun. After PBS wash, virus pellets were resuspended in PBS containing 0.2% SDS and 5 mM EDTA. Virus capsid protein was then quantified using Biorad protein assay kit and normalized to mock infected and concentrated samples.

### CVA21 antibody generation

Twenty female Balb/C mice were inoculated intraperitoneally with 1e6 CVA21 three times over a period of six weeks. Two weeks following the final inoculation, mice were terminally bled, serum was collected and pooled and frozen at −20°C for use in immunoblotting.

### Western blot analysis

Supernatant from miRNA-mimic timecourse experiments were sucrose-purified as above and run on a 12.5% SDS page gel and transferred to a PVDF membrane using the Trans-Blot SD semi-dry transfer apparatus (BioRad) for 45 minutes at 15 Volts. Blots were blocked in TBS-10% milk for 1 hour at room temperature. Primary CVA21 antibody generated as described above was diluted 1∶500 in TBS-5% milk +.05% Tween and blots were incubated overnight at 4°C. Membranes were washed in TBS +.1% Tween and incubated in secondary Goat-Anti Mouse IgG (Dako) at a 1∶5000 dilution in TBS-5% milk+.05%Tween at room temperature for 1 hour. Membranes were washed in TBS +.1% Tween and bound antibodies were detected using SuperSignal West Pico Chemluminescent reagent (Pierce).

### In vivo experiments

All animal protocols were reviewed and approved by Mayo Clinic Institutional Care and Use Committee. CB17 ICR-SCID mice were obtained from Harlan. Mice were irradiated and implanted with 5e6 Kas 6/1 or Mel 624 cells in the right flank. When tumors reached an average of .5×.5 cm, tumors were treated with 1e6 CVA21. Tumor volume was measured using a hand held caliper and blood was collected by retro-orbital bleeds.

## References

[1] Lewis BP, Burge CB, Bartel DP (2005). Conserved seed pairing, often flanked by adenosines, indicates that thousands of human genes are microRNA targets.. Cell.

[2] Griffiths-Jones S, Saini HK, van Dongen S, Enright AJ (2008). miRBase: tools for microRNA genomics.. Nucleic Acids Res.

[3] Huang Q, Gumireddy K, Schrier M, le Sage C, Nagel R (2008). The microRNAs miR-373 and miR-520c promote tumour invasion and metastasis.. Nat Cell Biol.

[4] Bartel DP (2004). MicroRNAs: genomics, biogenesis, mechanism, and function.. Cell.

[5] Brown BD, Gentner B, Cantore A, Colleoni S, Amendola M (2007). Endogenous microRNA can be broadly exploited to regulate transgene expression according to tissue, lineage and differentiation state.. Nat Biotechnol.

[6] Westerhout EM, Ooms M, Vink M, Das AT, Berkhout B (2005). HIV-1 can escape from RNA interference by evolving an alternative structure in its RNA genome.. Nucleic Acids Res.

[7] Gu S, Jin L, Zhang F, Sarnow P, Kay MA (2009). Biological basis for restriction of microRNA targets to the 3′ untranslated region in mammalian mRNAs.. Nat Struct Mol Biol.

[8] Pillai RS, Bhattacharyya SN, Artus CG, Zoller T, Cougot N (2005). Inhibition of translational initiation by Let-7 MicroRNA in human cells.. Science.

[9] Humphreys DT, Westman BJ, Martin DI, Preiss T (2005). MicroRNAs control translation initiation by inhibiting eukaryotic initiation factor 4E/cap and poly(A) tail function.. Proc Natl Acad Sci U S A.

[10] Barnes D, Kunitomi M, Vignuzzi M, Saksela K, Andino R (2008). Harnessing endogenous miRNAs to control virus tissue tropism as a strategy for developing attenuated virus vaccines.. Cell Host Microbe.

[11] Cawood R, Chen HH, Carroll F, Bazan-Peregrino M, van Rooijen N (2009). Use of tissue-specific microRNA to control pathology of wild-type adenovirus without attenuation of its ability to kill cancer cells.. PLoS Pathog.

[12] Ylosmaki E, Hakkarainen T, Hemminki A, Visakorpi T, Andino R (2008). Generation of a conditionally replicating adenovirus based on targeted destruction of E1A mRNA by a cell type-specific microRNA.. J Virol.

[13] Kelly EJ, Hadac EM, Greiner S, Russell SJ (2008). Engineering microRNA responsiveness to decrease virus pathogenicity.. Nat Med.

[14] Jopling CL, Yi M, Lancaster AM, Lemon SM, Sarnow P (2005). Modulation of hepatitis C virus RNA abundance by a liver-specific MicroRNA.. Science.

[15] Brown BD, Venneri MA, Zingale A, Sergi Sergi L, Naldini L (2006). Endogenous microRNA regulation suppresses transgene expression in hematopoietic lineages and enables stable gene transfer.. Nat Med.

[16] Gottwein E, Cullen BR (2008). Viral and cellular microRNAs as determinants of viral pathogenesis and immunity.. Cell Host Microbe.

[17] Sullivan CS, Grundhoff AT, Tevethia S, Pipas JM, Ganem D (2005). SV40-encoded microRNAs regulate viral gene expression and reduce susceptibility to cytotoxic T cells.. Nature.

[18] Pedersen IM, Cheng G, Wieland S, Volinia S, Croce CM (2007). Interferon modulation of cellular microRNAs as an antiviral mechanism.. Nature.

[19] Kelly EJ, Russell SJ (2009). MicroRNAs and the regulation of vector tropism.. Mol Ther.

[20] Schiff GM, Sherwood JR (2000). Clinical activity of pleconaril in an experimentally induced coxsackievirus A21 respiratory infection.. J Infect Dis.

[21] Dekel B, Yoeli R, Shulman L, Padeh S, Passwell JH (2002). Localized thigh swelling mimicking a neoplastic process: involvement of coxsackie virus type A21.. Acta Paediatr.

[22] Filipowicz W, Bhattacharyya SN, Sonenberg N (2008). Mechanisms of post-transcriptional regulation by microRNAs: are the answers in sight?. Nat Rev Genet.

[23] Petersen CP, Bordeleau ME, Pelletier J, Sharp PA (2006). Short RNAs repress translation after initiation in mammalian cells.. Mol Cell.

[24] Mathonnet G, Fabian MR, Svitkin YV, Parsyan A, Huck L (2007). MicroRNA inhibition of translation initiation in vitro by targeting the cap-binding complex eIF4F.. Science.

[25] Gentner B, Schira G, Giustacchini A, Amendola M, Brown BD (2009). Stable knockdown of microRNA in vivo by lentiviral vectors.. Nat Methods.

[26] Teterina NL, Zhou WD, Cho MW, Ehrenfeld E (1995). Inefficient complementation activity of poliovirus 2C and 3D proteins for rescue of lethal mutations.. J Virol.

[27] Van Der Velden A, Kaminski A, Jackson RJ, Belsham GJ (1995). Defective point mutants of the encephalomyocarditis virus internal ribosome entry site can be complemented in trans.. Virology.

[28] Jia XY, Van Eden M, Busch MG, Ehrenfeld E, Summers DF (1998). trans-encapsidation of a poliovirus replicon by different picornavirus capsid proteins.. J Virol.

[29] Etchison D, Milburn SC, Edery I, Sonenberg N, Hershey JW (1982). Inhibition of HeLa cell protein synthesis following poliovirus infection correlates with the proteolysis of a 220,000-dalton polypeptide associated with eucaryotic initiation factor 3 and a cap binding protein complex.. J Biol Chem.

[30] Rose JK, Trachsel H, Leong K, Baltimore D (1978). Inhibition of translation by poliovirus: inactivation of a specific initiation factor.. Proc Natl Acad Sci U S A.

[31] Wien MW, Chow M, Hogle JM (1996). Poliovirus: new insights from an old paradigm.. Structure.

[32] Ebert MS, Neilson JR, Sharp PA (2007). MicroRNA sponges: competitive inhibitors of small RNAs in mammalian cells.. Nat Methods.

